# Extending Non-Ambiguity Range of Dual-Comb Ranging for a Mobile Target Based on FPGA

**DOI:** 10.3390/s22186830

**Published:** 2022-09-09

**Authors:** Ruoyu Liu, Haoyang Yu, Yue Wang, Yu Li, Xinda Liu, Pengpeng Zhang, Qian Zhou, Kai Ni

**Affiliations:** 1 Division of Advanced Manufacturing, Tsinghua Shenzhen International Graduate School, Tsinghua University, Shenzhen 518055, China; 2 Department of Automation, Central South University, Changsha 410083, China

**Keywords:** dual-comb ranging, field-programmable gate array (FPGA), non-ambiguity range, dead-zone free, real-time system

## Abstract

Dual-comb ranging (DCR) is an important method in absolute distance ranging because of its high precision, fast acquisition rate, and large measuring range. DCR needs to obtain precise results during distance measurements for a mobile target. However, the non-ambiguity range (NAR) is a challenge when pushing the dual-comb ranging to the industry field. This paper presents a solution for extending NAR by designing an algorithm and realizing it on a field-programmable gate array (FPGA). The algorithm is robust when facing the timing jitter in the optical frequency comb. Without averaging, the Allan deviation of the results in 1 ms is ∼3.89 μm and the Allan deviation of the results is ∼0.37 μm at an averaging time of 100 ms when the target object is standstill near the NAR. In addition, several ranging experiments were conducted on a mobile target whose speed was from ∼5 mm/s to ∼10 mm/s. The experimental results verify the effectiveness and robustness of our design. The implemented design is an online and real-time data processing unit that shows great industrial potential for using the DCR system.

## 1. Introduction

Absolute distance measurement is necessary for the satellite formation flying and processes of precision manufacturing [1,2]. The invention of the optical frequency comb (OFC) provides a promising opportunity for precision absolute distance measuring [3]. The OFC contains a number of discrete modes in the frequency spectrum which are regularly spaced and the optical frequency $\nu_{n}$ of each mode is $f_{0}+nf_{r}$, where $f_{0}$ is the offset frequency and $f_{r}$ is the repetition frequency [4,5]. The offset frequency $f_{0}$ and repetition frequency $f_{r}$ are radio frequencies (rf) which means that the OFC can link the frequencies in the optical domain and frequencies in the radio domain. This feature facilitates the developments in many fields such as optical frequency synthesis [6,7,8], precision absorption spectroscopy [9,10,11,12,13], optical atomic clocks [14,15], microwave frequency transfer [16,17], and absolute distance measurement [3,18].

Dual-comb ranging (DCR) is based on the two fiber-based OFCs with slightly different repetition frequencies, acting as a signal comb and a local oscillator (LO) comb, respectively. Since the dual-comb method proposed by Schiller in 2002 for performing accurate spectral characterization of samples [19] and Coddington et al. in NIST applied dual-comb in measuring absolute distance in 2009 firstly [20], DCR draws great attention from academia and industry for its outstanding features [21,22,23,24]. DCR can take advantage of time-of-flight(TOF) and interferometric method together so that the measurement can be performed with high precision and fast update rates [20]. For the combining of the TOF and interferometric method, the measured absolute distance can be obtained from two parts. The first part is based on the time delay between the envelope of reference interferograms and target interferograms. The second part is provided from the carrier phase of the pulses.

DCR systems utilize the TOF principle from the perspective of the time domain and apply the multi-heterodyne principle from the frequency domain [22]. The precision of the DCR systems can satisfy the requirements of industry fields mostly. Performance and limitations of DCR systems involving electro-optic modulators are discussed in detail in [25]. In addition to combs with active stabilization, DCR with free-running dual-comb solid-state oscillators is also explored in [26] which can reach a resolution of 0.55 μm. A more detailed review of DCR can be found in [22]. The dead zones and non-ambiguity range during the measuring process are two of the main factors that restrict the application of DCR systems in long-distance measuring.

To remove the dead zones in the measurements, Lee et al. in KAIST used orthogonal polarization to separate the measurement pulses from the reference pulses [27]. This method proves the effectiveness of removing the dead zones using orthogonal polarization. Extending the non-ambiguity range mainly depends on creating a longer synthetic wavelength. In [20], it switches the OFC sources of the DCR system and applies the Vernier effect to obtain a larger non-ambiguity range. With an alternate sampling method combined polarization-multiplexed dual-comb fiber laser, the swap of OFC sources can be finished without manually [28]. Tuning the repetition frequency of the signal laser to extend the non-ambiguity range is also a common method [27,29], but the change of the frequency of the signal laser $\delta_{r}$ should be tuned carefully to avoid $L^{\prime }-L\approx 0$ so as to obtain integer *m* correctly. In addition to DCR systems, there are methods using triple combs to extend the non-ambiguity range [30,31].

In this paper, we propose an algorithm for determining the absolute distance of a mobile target without changing the repetition frequencies of the dual-combs and implement it on FPGA as a signal processing unit. Compared with digitizers based on a central processing unit (CPU), FPGA can run instructions in parallel and be flexibly programming configured to achieve high processing speed. Our algorithm fully takes advantage of the parallelism of FPGA and the signal processing unit can obtain the measured absolute distance in real-time by pipelining. Two OFCs were used in our experiments with repetition frequencies locked to an external reference only, and the offset frequencies were not locked. The target mirror moved back and forth near the non-ambiguity range $L_{\mathrm{NAR}}$ to verify the effectiveness of extending the non-ambiguity range when the target object moved beyond the $L_{\mathrm{NAR}}$. When the target object stays near the $L_{\mathrm{NAR}}$, the Allan deviation can achieve ∼3.89 μm in 1 ms and ∼0.37 μm in 100 ms. Our method is a real-time and online solution based on FPGA that can promote the application of the DCR system in the field environment.

## 2. Materials and Methods

**Figure 1:**
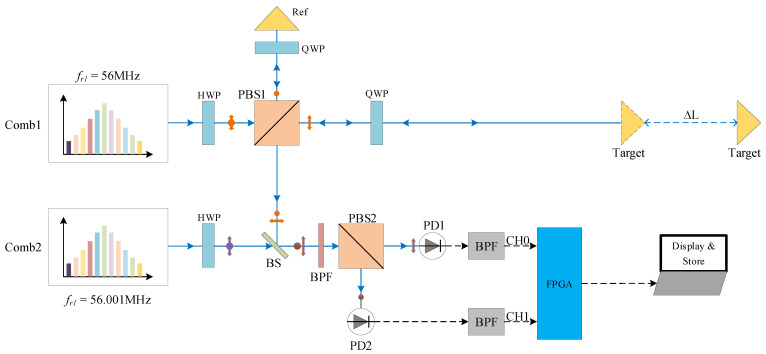
Experimental setup of the dual-comb ranging system. Blue solid line with arrow represents optical path, blue dotted line represents the moving range of the target mirror, black dotted line represents the electrical connection, while circle and double arrow represent orthogonal polarization states. The repetition frequencies of comb1 and comb2 are 56 MHz and 56.001 MHz. HWP: half-wave plate; QWP: quarter-wave plate; PBS: polarization beam splitter; Ref, Target: TIR retroreflector prism; BS: beam splitter; PD: photodetector; BPF: band-pass filter.

The experimental setup of the dual-comb ranging system is shown in Figure 1. The signal comb and LO comb were homemade to supply femtosecond pulses at ∼56 MHz repetition rate around 1560 nm center wavelength. The signal comb and the LO comb are locked to an external reference, whose repetition frequencies are $f_{r1}=56$ MHz and $f_{r2}=56.001$ MHz, and the Allan deviation of repetition frequencies is less than 270 μHz at 1 s gate time which means the stability of repetition frequencies is better than 5e-12@1 s. The locking bandwidth of the repetition rate phase locking is tens of hertz. The difference in repetition frequencies between signal comb and LO comb is $\Delta f_{r}=f_{r1}-f_{r2}=1000$ Hz. Polarization beam splitters (PBS) whose extinction ratio is bigger than 1000 and quarter-wave plates (QWP) are used together to avoid dead zones since the reference interferograms and measurement interferograms are separated. Before PBS2, an optical band-pass filter (BPF; FWHM 6nm at 1550 nm) is used to meet the Nyquist sampling condition [32]. Signals from the reference mirror and the measurement target will incident into two individual photodetectors (1811-FC, Newport, Santa Clara, CA, USA).The output of these two photodetectors is connected to the input of two electronic band-pass filters (2~20 MHz band-pass frequency), respectively. The analog electrical signals from BPF are the input signals of channel 0 and channel 1 for our homemade FPGA board (Zynq-XC7Z020). The input signals of these two channels will be sampled by two 14-bit analog-to-digital converters (ADC). These two ADCs (ADS4145, Texas Instruments, Dallas, TX, USA) are embedded on the FPGA board as a peripheral.After the data was processed by FPGA, the results of distance can be transmitted to the PC for displaying and storing via a universal asynchronous receiver transmitter (UART).

The signal comb emits pulses which are directed to the reference mirror and target mirror with orthogonal polarization states. These pulses cannot be detected by standard photodetectors, which do not have enough bandwidth for femtosecond pulses. With the pulses from LO comb samples signals that are reflected from the reference mirror and target mirror, then the effect of the multi-heterodyne principle in the field of dual-comb spectroscopy [33] allows the modes in the optical frequency regime to down-convert to the RF domain which can be detected by standard photodetectors. The difference in repetition frequencies permits the LO comb to finish an equivalent sampling process [34]. This sampling process generates two interferograms: reference interferograms which are produced by signals reflected from the reference mirror, and measurement interferograms which are produced by signals from the target mirror.

**Figure 2:**
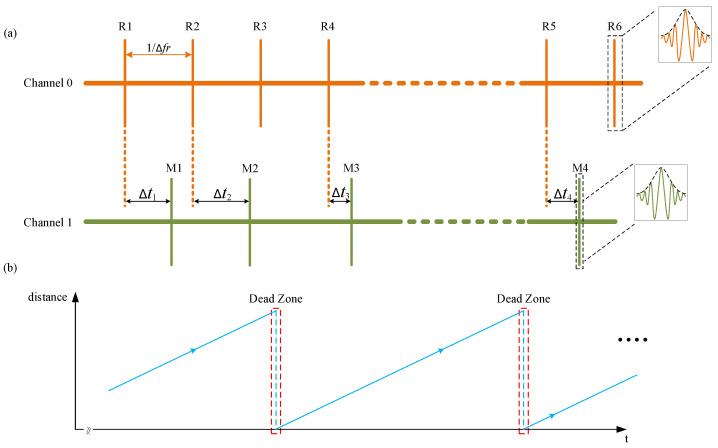
Schematic of the measurement procedure: (**a**) Input signals of two channels. R peaks represent reference interferograms (orange) and M peaks represent measurement interferograms (green). The local enlarged figure of R1 and M1 shows the broadened signals which are detected by photodetectors and digitized by ADC. The black dotted line represents the envelope of the interferograms which is Gaussian shape. The delayed time Δt between paired R peaks and M peaks is changing as target mirror moving away. (**b**) Relationship between measured distance and measuring time when target mirror moving away at the same speed.

The signals after the equivalent sampling process in the time domain are shown in Figure 2a, which are also the input signals of the FPGA board. Reference interferograms are denoted by R peaks and measurement interferograms are denoted as M peaks.

There are two input channels on our FPGA board. As shown in Figure 2a, R peaks are input signals of channel 0 and M peaks are input signals of channel 1. The local enlarged figures of Figure 2a show detailed reference interferograms and measurement interferograms. R peaks will appear periodically, and the period is $1/\Delta f_{r}=1$ ms which is called update period $T_{\mathrm{update}}$. Only when the target mirror stands still, then will the M peaks update periodically with the period equal to $1/\Delta f_{r}$. Otherwise, the update period $T_{\mathrm{update}}$for M peaks is not fixed. $\Delta t_{1}$ in Figure 2a is the time delay between paired R peaks and M peaks. If there is one M peak in two adjacent R peaks, this situation is called the RMR event and the M peak is paired with the first R peak in the RMR event. The absolute distance $L_{\mathrm{abs}}$ is calculated as

(1) $$L_{\mathrm{abs}}=n\cdot L_{\mathrm{NAR}}+D,$$

where *n* is a positive integer with $L_{\mathrm{NAR}}$ is the non-ambiguity, and *D* is the distances to be measured within the given non-ambiguity range $L_{\mathrm{NAR}}$. Non-ambiguity range $L_{\mathrm{NAR}}$ is equal to $c/2n_{g}f_{r}$,where *c* is the speed of light in vacuum, $n_{g}$ is the group refractive index of air, and $f_{r}$ is the repetition frequency of the signal comb. Measured distance *D* within the non-ambiguity range $L_{\mathrm{NAR}}$ can be determined by the delayed time Δt between paired R peaks and M peaks in RMR event [20,35].

Due to the update period $T_{\mathrm{update}}$ of reference interferograms is equal to $1/\Delta f_{r}$, and the non-ambiguity range $L_{\mathrm{NAR}}$ is calculated by $c/2f_{r}$, then the absolute distance $L_{\mathrm{abs}}$ can be derived by

(2) $$L_{\mathrm{abs}}=\left(n+\frac{\Delta t}{T_{\mathrm{update}}}\right)\cdot L_{\mathrm{NAR}}.$$

In order to obtain the measured distance, it is necessary to obtain the value of positive integer *n* and delayed time Δt.

Figure 2b shows the relationship between measured distance and measuring time when the target is moving away at the same speed. As the target mirror goes away, the measured distance within the non-ambiguity range increases. In Figure 2a, delayed time $\Delta t_{2}$ is bigger than $\Delta t_{1}$ as the target mirror is moving away. So the ratio between delayed time Δt and update period $T_{\mathrm{update}}$ is growing gradually. Once the measured absolute distance is equal to one non-ambiguity range $L_{\mathrm{NAR}}$ or multiple non-ambiguity range $k\cdot L_{\mathrm{NAR}}$, R peaks and M peaks align temporally. This situation leads to dead zones in the traditional dual-comb range, because R peaks and M peaks are overlapped in the same input channel [24]. As the target mirror continues to move, the R peaks no longer align with the M peaks and the delayed time Δt between paired R peaks and M peaks starts to increase from 0. Then, the delayed time Δt changes as the same pattern as the target mirror has not crossed the dead zone before. So the measured distance is wrapped after the target mirror crosses the dead zone as Figure 2b shows.

Figure 2b illustrates that the relationship between measured distance and measuring time is linear within the non-ambiguity range which indicates the velocity of the target mirror is constant during the whole moving process. When the target mirror is crossing the dead zone, the measured distance is wrapped which means the velocity derived from the measured distance and measuring time is changing severely at this moment. Suppose that the difference in measured distance between two adjacent update periods is $v_{\mathrm{adj}}$ which can be expressed by

(3) $$v_{\mathrm{adj}}=\frac{D_{\mathrm{cs}}-D_{\mathrm{ls}}}{T_{\mathrm{update}}},$$

where $D_{\mathrm{cs}}$ is the measured distance in the current update period within the $L_{\mathrm{NAR}}$ and $D_{\mathrm{ls}}$ is the measured distance in the last update period within the $L_{\mathrm{NAR}}$. Normally when the target mirror moves within the $L_{\mathrm{NAR}}$, $v_{\mathrm{adj}}$ changes in a very small range due to the relatively high update rate of results compared with the speed of the target mirror. Once $v_{\mathrm{adj}}$ changes to a very large value which is close to $L_{\mathrm{NAR}}$, that means the target mirror is crossing the dead zone.

**Figure 3:**
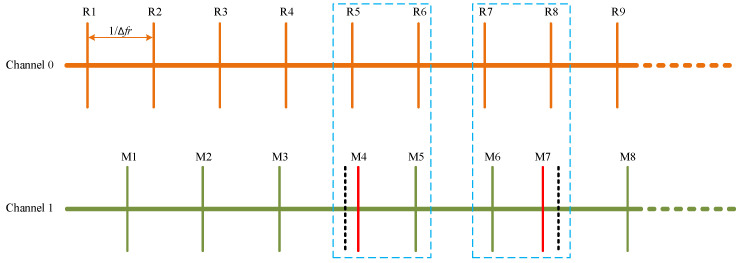
Abnormal situations appear due to the timing jitter in the combs when the target mirror is crossing dead zones. The blue dotted frames indicate there are abnormal situations appear. The black dotted line represents where the M peaks are supposed to be and the solid red line represents where the M peaks are actually. Solid orange lines and solid green lines represent interferograms without timing jitter.

However, the timing jitter in the OFCs will lead to abnormal situations when the target mirror is near the $k\cdot L_{\mathrm{NAR}}$ (k=1,2,3...). The timing jitter in the OFCs will scale up by a factor of $f_{r}/\Delta f_{r}$ which is caused by asynchronous optical sampling [36] so that the timing jitter will bring obvious influence on measuring results. As shown in Figure 3, there are two M peaks between adjacent R peaks in blue dotted frames when the target mirror is crossing the dead zones positively which means the measured absolute distance should increase gradually. The situation presented in the blue dotted frame is called the RMMR event. RMMR event appears as R peaks and M peaks are going to align temporally and the timing jitter changes the positions of peaks. There should be no RMMR event when the target mirror moves away if there is no timing jitter, but when the target mirror moves back, one RMMR exists as R peaks and M peaks are about to align temporally even if there is no timing jitter during the whole process. However, the real situations with the timing jitter are more complicated than the ideal scenario.

Technically, it is not distinguishable for only one normal RMMR event from other abnormal RMMR phenomena caused by timing jitter when the target mirror is crossing the dead zones negatively. The proposed algorithm is robust in that it still can obtain the value of absolute distance with precision when under such complex real situations.

Given there is only one RMMR event when the target mirror moves back in the ideal scenario, the algorithm only outputs one of two M peaks in RMMR events. RMMR event means that there are two results normally, but the algorithm only outputs one which means it will lose one result in thousands of results. It brings consistency by sacrificing only one result out of thousands, which makes it easier for the abstractions of the algorithm.

**Figure 4:**
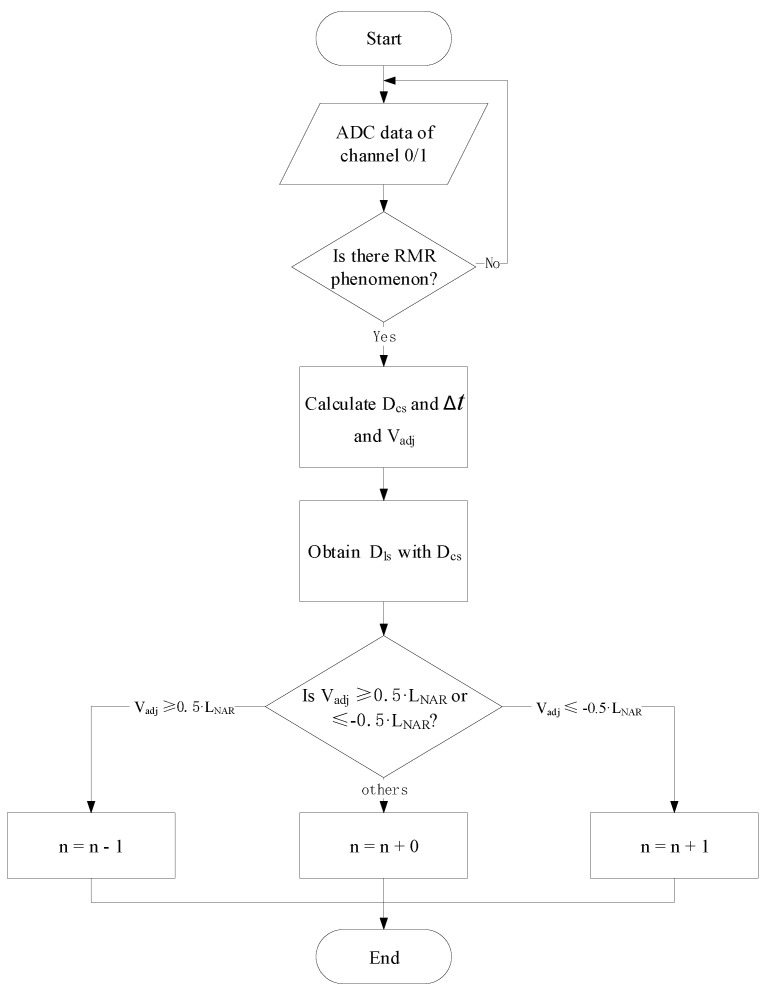
The algorithm that determines positive integer *n*. Input data are from channel 0 and channel 1. RMR: two adjacent R peaks with one M peak between them. $D_{\mathrm{cs}}$: measured distance within $L_{\mathrm{NAR}}$ at current state.$D_{\mathrm{ls}}$: measured distance within $L_{\mathrm{NAR}}$ at last state. $V_{\mathrm{adj}}$: is the difference between $D_{\mathrm{cs}}$ and $D_{\mathrm{ls}}$.

The timing jitter also leads to difficulty in determining positive integer *n* when the target mirror is near the dead zones. The algorithm for determining *n* is illustrated by Figure 4. After the whole system starts to run, two ADCs convert the input analog electrical signals from channel 0 and channel 1 to digital signals. Only when an RMR event arises, then will FPGA begin to calculate measured distance within $L_{\mathrm{NAR}}$ and $V_{\mathrm{adj}}$.

After obtaining the value of $V_{\mathrm{adj}}$, compare it with half of the $L_{\mathrm{NAR}}$ and half of the $-L_{\mathrm{NAR}}$. If $V_{\mathrm{adj}}⩾0.5\cdot L_{\mathrm{NAR}}$, then positive integer *n* should decrease by one. If $V_{\mathrm{adj}}⩽-0.5\cdot L_{\mathrm{NAR}}$, then positive integer *n* should increase by one. In other situations, the positive integer *n* should remain the value.

**Figure 5:**
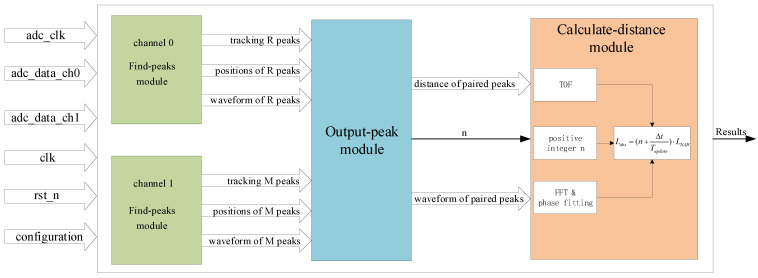
Block diagram of signal processing unit implemented on FPGA board. The signal processing unit consists of four sub-modules. The function of the find-peaks module is finding the reference interferograms from channel 0 and the measurement interferograms from 1. The output-peak module is responsible for giving the necessary values to finish calculating the absolute distance. The calculate-distance module performs the final calculation of absolute distance.

Besides positive integer *n*, time delay Δt between paired R peaks and M peaks is also an essential variable for calculating absolute distance $L_{\mathrm{abs}}$. In order to obtain the value of Δt as accurately as possible, there are two parts for calculating Δt. One part is the integer part which is obtained from find-peaks modules, the other part is the fractional part which is obtained from the calculate-distance module as depicted in Figure 5.

Figure 5 shows the block diagram of the signal processing unit which was implemented on the FPGA board to obtain the value of positive integer *n* and measured distance *D*. The output results of the signal processing unit are the absolute distance $L_{\mathrm{abs}}$.

The input signals of the signal processing unit are:

1. clk: system clock whose frequency is 125 MHz;
2. rst_n: asynchronous reset signal for the whole system;
3. adc_clk: clock of ADC whose frequency is 100 MHz;
4. adc_data_ch0: digital signal from ADC of channel 0;
5. adc_data_ch1: digital signal from ADC of channel 1;
6. configuration: general configuration information, e.g., the repetition frequencies of two combs.

The signal processing unit is composed of four sub-modules. The find-peaks module is designed to find R peaks and M peaks in channel 0 and channel 1 with an adaptive sliding window [37] and to synchronize the input data in the ADC clock domain to the system clock domain so as to satisfy the timing request. The find-peaks module outputs the positions of the peaks and the waveform of the peaks. In order to improve the robustness of the design, the find-peaks module also outputs signals for tracking peaks so that it can filter out fake peaks. Moreover, the information of tracking can help determine whether the input R peaks and M peaks are paired and whether two R peaks are adjacent or not.

The output-peak module implements the algorithm of extending the non-ambiguity range as shown in Figure 4. It calculates the distance between paired R peaks and M peaks in the sample points unit so that the integer part of the Δt in Equation (2) can be obtained by combining the sampling rate of ADC. Furthermore, the output-peak module will calculate the positive integer *n* according to the algorithm described in Figure 4. The output-peak module outputs the waveform of paired peaks and the distance between paired R peaks and M peaks, and the positive integer *n* as denoted by *n* in Figure 5. The calculate-distance module will perform a fast Fourier transform (FFT) and phase fitting as illustrated in [20,22] to obtain the fractional part of delay time Δt. In addition, the calculate-distance module will calculate the final value of the absolute distance with the positive integer *n* and delayed time Δt, then output results.

## 3. Results

After finishing the preliminary behavioral simulation in QUESTASIM, practical experiments were conducted. The setup of the measuring system is demonstrated in Figure 1. The target mirror was fixed on the moving table of a slide rail which permits measuring the distance regardless of whether the target mirror is standstill or moving. The velocity can be controlled by the controller of the stepper motor which was installed on the slide rail.

**Figure 6:**
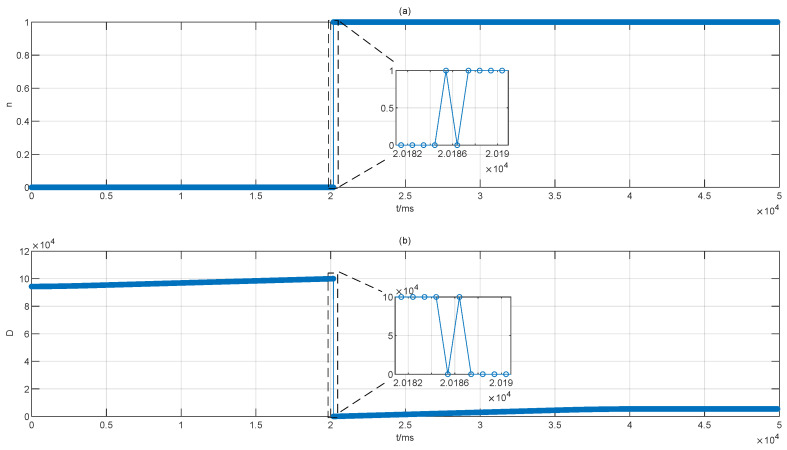
Effectiveness of the proposed signal processing unit. The target mirror is moving at the same speed. (**a**) Value of positive integer *n* which counts for the number of the non-ambiguity range. In the code, the positive integer *n* was denoted as *n*. (**b**)The distance between the paired R peaks and m peaks which are stored in RAM. This value was denoted as *D* in the code.

The effectiveness of the proposed signal processing unit is shown in Figure 6 when the target mirror is moving at about 8.125 mm/s for about 30 cm. As shown in Figure 6a, the *n* given by the output-peak module counts for the number of the non-ambiguity range will change from 0 to 1 as the target mirror is crossing the dead zone positively. However, there was a fluctuation when the value of the positive integer *n* changed from 0 to 1 on account of timing jitter.The distance between paired R peaks and M peaks determined by the output-peak module was denoted as *D* as shown in Figure 6b. The unit of distance is the sample points of ADC, and the distance is growing at the identical speed with the move of the target mirror within the non-ambiguity range $L_{\mathrm{NAR}}$.

However, the *D* was wrapped when the target mirror was crossing the dead zone, and there was also a fluctuation in the value of *D* which was complementary to the fluctuation in the *n*. Therefore, there will be no fluctuation in the results of measured absolute distance when combining the *n* and the value of *D*.

**Figure 7:**
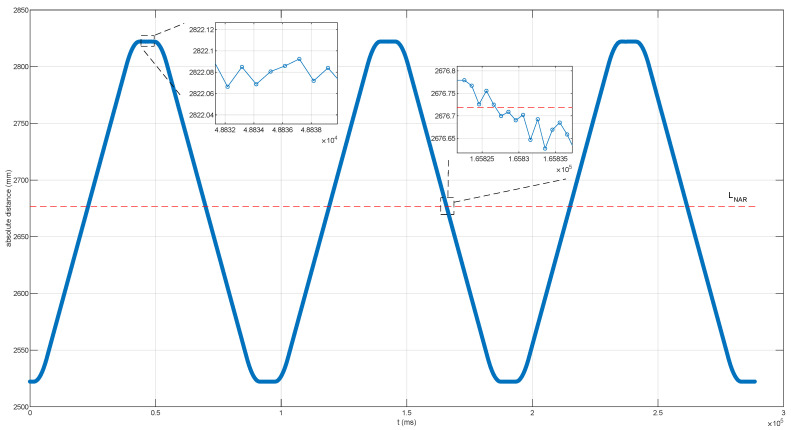
The blue line represents the results of measurements in the whole process when the target mirror moved back and forth at about 8.125 mm/s for about 30 cm. The absolute distance that the red dotted line represents is the non-ambiguity range $L_{\mathrm{NAR}}$, which is 2676.718375 mm in our DCR system.

With the help of the *n* and *D*, the final absolute distance measurement can be obtained when the target mirror moved back and forth as Figure 7 illustrates.

The target mirror moved away at ∼8.125 mm/s for about 30 cm at first, and standstill for ∼5 ms, then went back at ∼8.125 mm/s for about 30 cm. The target mirror moved back and forth three times in all as the blue line shown in Figure 7. The local enlarged figure of Figure 7 shows the signal processing unit designed can obtain the absolute distance with precision no matter whether the target mirror stayed beyond one $L_{\mathrm{NAR}}$ or crossed the dead zones negatively, and the results were with a slight ripple caused by residual timing jitter in the combs. We conducted a series of experiments with the target mirror moving at different speeds which were in the range of 5 mm/s to 10 mm/s given the limitation of the slide rail. The results of these experiments were similar to Figure 7, and the slope of the movement curve was the difference among these curves of results which agreed with the different setting velocity of the target mirror. Due to the repetition frequency of the signal comb being 56 MHz, the non-ambiguity range $L_{\mathrm{NAR}}$ is ∼2676.718375 mm as the red dotted line depicted in Figure 7. It was thanks to the coordination between the optical design and the proposed signal processing unit that there were no dead zones and the non-ambiguity range was extended during the whole measuring process of our experiments.

**Figure 8:**
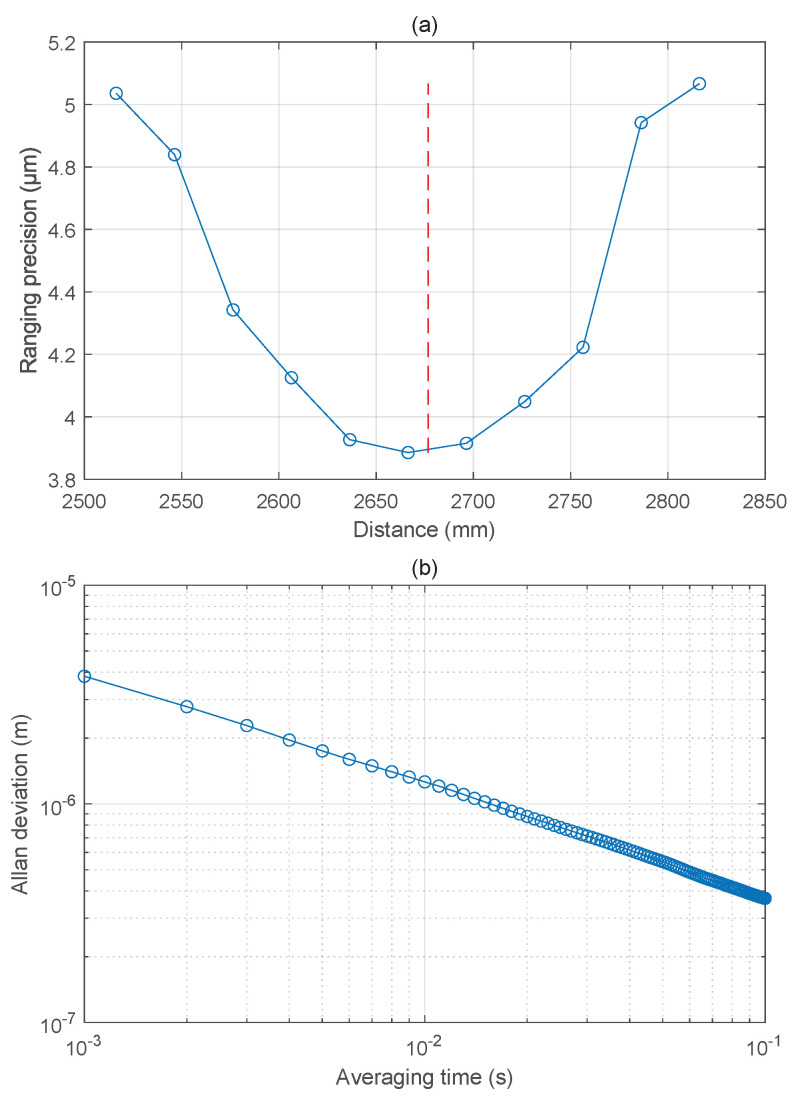
The precision of the experimental results. (**a**) The relationship between the ranging precision and distance. The red dot line represents the non−ambiguity range $L_{\mathrm{NAR}}$. (**b**) The Allan deviation when the target mirror was fixed near the $L_{\mathrm{NAR}}$ at different averaging times. The Allan deviation is about 3.89 μm at averaging time of 1 ms and about 0.37 μm at averaging time of 100 ms.

After getting the right *n*, we also obtained the relationship between the measured distance and ranging precision as presented in Figure 8a. The target mirror was fixed at different positions. The signals were sampled for about 10,000 ms so that there were approximately 10,000 results of measurements for each position. In Figure 8a, the ranging precision is converging as the target mirror moves towards the $L_{\mathrm{NAR}}$ and the ranging precision is diverging as the target mirror moves away from the $L_{\mathrm{NAR}}$. Figure 8b shows the Allan deviation at different averaging times from 1 ms to 100 ms when the target mirror was fixed near the $L_{\mathrm{NAR}}$. Without averaging, the Allan deviation in 1 ms is 3.89 μm at the position near the $L_{\mathrm{NAR}}$ as shown in Figure 8b. After 100 ms averaging, the Allan deviation can reach about 0.37 μm. The precision is determined by the timing jitter of the dual-comb system [36,38,39,40], which can be further improved by introducing multi-pulse consequence [41,42].

Due to the proposed signal processing unit taking advantage of the feature that each module runs in parallel in FPGA, results of measurements can be obtained for each update period $T_{\mathrm{update}}$ in real-time as long as the signals have a good signal-noise-ratio(SNR).

## 4. Discussion

An algorithm for extending the non-ambiguity range in the DCR system is proposed and implemented on the FPGA platform as a robust signal processing unit. There are two key points in designing the algorithm shown in Figure 4, one is detecting RMR events for determining the R peaks and M peaks are paired or not, the other is the sharp change in target mirror speed for detecting whether the target mirror is crossing the dead zone. The proposed algorithm can handle the situations when the target mirror moves more than $k\cdot L_{\mathrm{NAR}}$ (k=1,2,3...) as long as the following two requirements are satisfied. The first requirement is for $V_{\mathrm{adj}}$. The maximum value of normal $V_{\mathrm{adj}}$ should not be greater than half of the $L_{\mathrm{NAR}}$, because half of the $L_{\mathrm{NAR}}$ is the threshold for counting *n* correctly as shown in Figure 4. The second requirement is for the SNR of the interferograms. The peak-to-peak values of the reference and measurement interferograms should be 40 mv at least for a good SNR to perform measurements correctly. The algorithm is implemented on FPGA as a signal processing unit.

The signal processing unit can obtain the value of absolute distance even though the OFCs have timing jitter which will lead to the fluctuation of *n* and *D* as shown in Figure 6. The fluctuations of these two variables are complementary, and the final calculated results of absolute distance will be reasonable when the target mirror is crossing the dead zones. The designed signal processing unit can achieve the requirements of high-precision and online and real-time when the target mirror is moving. In addition, the signal processing unit is able to work correctly when the target mirror moves back and forth for about 4 h continuously, which means the signal processing unit is robust.

By the use of the signal processing unit, the Allan deviation in 1 ms was 3.89 μm without averaging while the non-ambiguity range $L_{\mathrm{NAR}}$ was about 2676.718375 mm on account of the repetition frequency of the signal comb is 56 MHz. After 100 ms averaging, the Allan deviation is about 0.37 μm. So the precision of the results can be improved by averaging results [25]. and leveraging the carrier phases of the interferograms to achieve a better precision [22]. Moreover, the change of precision when the target mirror stayed at different positions is agreed with the relationship between ranging precision and target mirror positions mentioned in [36] which only discusses the effects introduced by timing jitter. That is to say, the precision of results will not get worse continuously as the target mirror moves beyond one non-ambiguity range.

However, a critical factor that should be taken carefully in our solution is the consistency of channels. Because of using polarization optics in our optical setup, there are two optical channels for measurement interferograms and reference interferograms, respectively. Naturally, there are two electrical input channels on the FPGA for these two optical interferograms.

For measurement interferograms and reference interferograms from different optical channels, the intensity of two interferograms should be close to each other. This goal can be achieved by adjusting the HWP after OFCs in the experimental setup. In addition, many factors in the electrical system can cause differences between the two electrical input channels such as the differences between these two photodetectors, the length of connecting wires and the routes on PCB, etc. In short, we should adopt symmetrical designs to try our best for keeping the consistency of the electrical parts before the ADCs. Otherwise, the analog electrical parts will lead to huge systematic errors. The differences caused by digital parts after ADCs will be relatively smaller compared with the analog electrical parts.

The proposed signal processing unit implemented on our homemade FPGA is meaningful to realize long-distance measuring with the DCR system for a mobile object. Furthermore, it is significant for accelerating the use of DCR in industrial applications such as self-driving.

## Data Availability

Not applicable.

## References

[1] Bosse H., Wilkening G. (2005). Developments at PTB in Nanometrology for Support of the Semiconductor Industry. Meas. Sci. Technol..

[2] Lim H.C., Bang H. (2009). Adaptive Control for Satellite Formation Flying under Thrust Misalignment. Acta Astronaut..

[3] Fortier T., Baumann E. (2019). 20 Years of Developments in Optical Frequency Comb Technology and Applications. Commun. Phys..

[4] Cundiff S.T., Ye J. (2003). Colloquium: Femtosecond Optical Frequency Combs. Rev. Mod. Phys..

[5] Udem T., Holzwarth R., Hänsch T. (2002). Optical Frequency Metrology. Nature.

[6] Giorgetta F.R., Coddington I., Baumann E., Swann W.C., Newbury N.R. (2010). Fast High-Resolution Spectroscopy of Dynamic Continuous-Wave Laser Sources. Nat. Photonics.

[7] Jost J.D., Hall J.L., Ye J. (2002). Continuously Tunable, Precise, Single Frequency Optical Signal Generator. Opt. Express.

[8] Spencer D.T., Drake T., Briles T.C., Stone J., Sinclair L.C., Fredrick C., Li Q., Westly D., Ilic B.R., Bluestone A. (2018). An Optical-Frequency Synthesizer Using Integrated Photonics. Nature.

[9] Ideguchi T., Poisson A., Guelachvili G., Picqué N., Hänsch T.W. (2014). Adaptive Real-Time Dual-Comb Spectroscopy. Nat. Commun..

[10] Ycas G., Giorgetta F.R., Baumann E., Coddington I., Herman D., Diddams S.A., Newbury N.R. (2018). High-Coherence Mid-Infrared Dual-Comb Spectroscopy Spanning 2.6 to 5.2 Mm. Nat. Photonics.

[11] Picqué N., Hänsch T.W. (2019). Frequency Comb Spectroscopy. Nat. Photonics.

[12] Yu H., Zhou Q., Li X., Wang X., Wang X., Ni K. (2021). Improving Resolution of Dual-Comb Gas Detection Using Periodic Spectrum Alignment Method. Sensors.

[13] Yu H., Li Y., Ma Q., Zhou Q., Li X., Ren W., Ni K. (2022). A Coherent-Averaged Dual-Comb Spectrometer Based on Environment-Shared Fiber Lasers and Digital Error Correction. Opt. Laser Technol..

[14] Diddams S.A., Udem T., Bergquist J.C., Curtis E.A., Drullinger R.E., Hollberg L., Itano W.M., Lee W.D., Oates C.W., Vogel K.R. (2001). An Optical Clock Based on a Single Trapped ^199^ Hg ^+^ Ion. Science.

[15] Papp S.B., Beha K., Del’Haye P., Quinlan F., Lee H., Vahala K.J., Diddams S.A. (2014). Microresonator Frequency Comb Optical Clock. Optica.

[16] Marra G., Margolis H.S., Lea S.N., Gill P. (2010). High-Stability Microwave Frequency Transfer by Propagation of an Optical Frequency Comb over 50 Km of Optical Fiber. Opt. Lett..

[17] Marra G., Slavík R., Margolis H.S., Lea S.N., Petropoulos P., Richardson D.J., Gill P. (2011). High-Resolution Microwave Frequency Transfer over an 86-Km-Long Optical Fiber Network Using a Mode-Locked Laser. Opt. Lett..

[18] Jang Y.S., Kim S.W. (2018). Distance Measurements Using Mode-Locked Lasers: A Review. Nanomanufacturing Metrol..

[19] Schiller S. (2002). Spectrometry with Frequency Combs. Opt. Lett..

[20] Coddington I., Swann W.C., Nenadovic L., Newbury N.R. (2009). Rapid and Precise Absolute Distance Measurements at Long Range. Nat. Photonics.

[21] Zhou S., Le V., Xiong S., Yang Y., Wu G. (2020). Dual-Comb Spectroscopy Resolved Three-Degree-of-Freedom Sensing. Photonics Res..

[22] Zhu Z., Wu G. (2018). Dual-Comb Ranging. Engineering.

[23] Han S., Kim Y.J., Kim S.W. (2015). Parallel Determination of Absolute Distances to Multiple Targets by Time-of-Flight Measurement Using Femtosecond Light Pulses. Opt. Express.

[24] Liu T.A., Newbury N.R., Coddington I. (2011). Sub-Micron Absolute Distance Measurements in Sub-Millisecond Times with Dual Free-Running Femtosecond Er Fiber-Lasers. Opt. Express.

[25] Martin B., Martin B., Feneyrou P., Dolfi D., Martin A. (2022). Performance and Limitations of Dual-Comb Based Ranging Systems. Opt. Express.

[26] Nürnberg J., Willenberg B., Phillips C.R., Keller U. (2021). Dual-Comb Ranging with Frequency Combs from Single Cavity Free-Running Laser Oscillators. Opt. Express.

[27] Lee J., Han S., Lee K., Bae E., Kim S., Lee S., Kim S.W., Kim Y.J. (2013). Absolute Distance Measurement by Dual-Comb Interferometry with Adjustable Synthetic Wavelength. Meas. Sci. Technol..

[28] Liu Y., Zhu Z., Yang J., Hu G. (2020). Fast Distance Measurement with a Long Ambiguity Range Using a Free-Running Dual-Comb Fiber Laser. Frontiers in Optics.

[29] Zhang H., Wei H., Wu X., Yang H., Li Y. (2014). Absolute Distance Measurement by Dual-Comb Nonlinear Asynchronous Optical Sampling. Opt. Express.

[30] Li T., Zhao X., Chen J., Yang J., Li Q., Li Y., Zheng Z. Absolute Distance Measurement with a Long Ambiguity Range Using a Tri-Comb Mode-Locked Fiber Laser. Proceedings of the 2019 Conference on Lasers and Electro-Optics (CLEO).

[31] Zhao X., Qu X., Zhang F., Zhao Y., Tang G. (2018). Absolute Distance Measurement by Multi-Heterodyne Interferometry Using an Electro-Optic Triple Comb. Opt. Lett..

[32] Coddington I., Swann W., Newbury N. (2010). Coherent Dual-Comb Spectroscopy at High Signal-to-Noise Ratio. Phys. Rev. A.

[33] Coddington I., Newbury N., Swann W. (2016). Dual-Comb Spectroscopy. Optica.

[34] Jiang Y., Liu Q., Cao H., Song Y. (2020). Peak Detection Based on FPGA Using Quasi-Newton Optimization Method for Femtosecond Laser Ranging. IEEE Access.

[35] Wu G., Zhou Q., Shen L., Ni K., Zeng X., Li Y. (2014). Experimental Optimization of the Repetition Rate Difference in Dual-Comb Ranging System. Appl. Phys. Express.

[36] Shi H., Song Y., Liang F., Xu L., Hu M., Wang C. (2015). Effect of Timing Jitter on Time-of-Flight Distance Measurements Using Dual Femtosecond Lasers. Opt. Soc. Am..

[37] Ni K., Dong H., Zhou Q., Xu M., Li X., Wu G. Interference Peak Detection Based on FPGA for Real-Time Absolute Distance Ranging with Dual-Comb Lasers. Proceedings of the International Conference on Optical Instruments and Technology.

[38] Yu H., Ni K., Zhou Q., Li X., Wang X., Wu G. (2019). Digital Error Correction of Dual-Comb Interferometer without External Optical Referencing Information. Opt. Express.

[39] Paschotta R., Schlatter A., Zeller S., Telle H., Keller U. (2006). Optical Phase Noise and Carrier-Envelope Offset Noise of Mode-Locked Lasers. Appl. Phys. B Lasers Opt..

[40] Yu H., Zhou Q., Li X., Wang X., Ni K. (2021). Mode-Resolved Dual-Comb Spectroscopy Using Error Correction Based on Single Optical Intermedium. Opt. Express.

[41] Yu H., Qian Z., Li X., Wang X., Ni K. (2021). Phase-Stable Repetition Rate Multiplication of Dual-Comb Spectroscopy Based on a Cascaded Mach–Zehnder Interferometer. Opt. Lett..

[42] Zhou S., Lin C., Yang Y., Wu G., Wu G. (2020). Multi-Pulse Sampling Dual-Comb Ranging Method. Opt. Express.

